# P-fimbriae in the presence of anti-PapA antibodies: new insight of antibodies action against pathogens

**DOI:** 10.1038/srep03393

**Published:** 2013-12-02

**Authors:** Narges Mortezaei, Bhupender Singh, Esther Bullitt, Bernt Eric Uhlin, Magnus Andersson

**Affiliations:** 1 Department of Physics, Umeå University, SE-901 87 Umeå, Sweden; 2 The Laboratory for Molecular Infection Medicine Sweden (MIMS) and Department of Molecular Biology, Umeå University, SE-901 87 Umeå, Sweden; 3 Department of Physiology and Biophysics, Boston University School of Medicine, Boston MA 02118-2526, USA; 4 These authors contributed equally to this work.

## Abstract

Uropathogenic strains of *Escherichia coli* establish urinary tract infections by attaching to host epithelial cells using adhesive organelles called fimbriae. Fimbriae are helix-like structures with a remarkable adaptability, offering safeguarding for bacteria exposed to changing fluid forces in the urinary tract. We challenged this property of P-fimbriae by cross-linking their subunits with shaft-specific antibodies and measuring the corresponding force response at a single organelle level. Our data show compromised extension and rewinding of P-fimbriae in the presence of antibodies and reduced fimbrial elasticity, which are important properties of fimbriae contributing to the ability of bacteria to cause urinary tract infections. The reduced elasticity found by cross-linking fimbrial subunits could thus be another assignment for antibodies; in addition to marking bacteria as foreign, antibodies physically compromise fimbrial function. We suggest that our assay and results will be a starting point for further investigations aimed at inhibiting sustained bacterial adhesion by antibodies.

Urinary tract infections (UTIs) are by far one of the most common pathological conditions requiring medical attention, with uropathogenic strains of the bacterium *Escherichia coli* (UPEC) being predominant etiological agents. UPEC are armed with micrometer long adhesion organelles known as fimbriae that exhibit sophisticated mechanical properties. Studies aimed to understand fimbrial mechanical properties at the single organelle level revealed their outstanding ability to withstand external force due to their remarkable degree of flexibility. Fimbriae are essential for bacterial adhesion to epithelia of urinary bladders and nephrons. Typical host reactions to bacterial UTI are a physiological response such as an increased urine flow resulting in an increased shear force acting on attached bacteria, and an eventually active immune response including production of antibodies^1^. It has been shown that the fimbrial shaft, with its quaternary structure often helical, is essential for sustained adhesion of bacteria against shearing forces that may occur in the urinary tract and in the intestinal tract^2^,^3^,^4^,^5^,^6^. A demonstration of this adhesive capacity is the binding via CFA/I pili of enterotoxigenic *E. coli* to erythrocytes. Bacteria that produced thin fibrillar structures that are incapable of coiling, as a result of a point mutation, could bind but could not sustain the attachment when exposed to shear forces^7^,^8^. Thus, a fully functional fimbrial shaft is important for bacterial attachment in environments where fluid flow is dynamic. It is therefore feasible that molecules or complexes that interact by binding to the shaft could interfere with its dynamic properties and make fimbriae dysfunctional, resulting in decreased attachment capabilities of bacteria. Recently the concept of possible interference with such compliance of fimbriae was tested with P-fimbriae, and a protein interacting with the major subunit was shown to impair the recoiling after a forced uncoiling^9^.

Humoral and secreted antibodies are known to play a role in the host defense against bacterial infections including UTIs. The presence of secretory antibodies was shown in the urothelium in response to UTIs^10^,^11^,^12^,^13^. It has been speculated that these antibodies can prevent establishment of a successful infection by interfering with bacterial adhesion^14^,^15^, a concept that has been utilized for development of a vaccine against bacterial adhesins to prevent UTIs^16^,^17^,^18^,^19^. In addition, the presence of antibodies against P-fimbriae has been reported in serum and urine of bacterial UTIs cases^20^,^21^,^22^. The role of antibodies in opsonization is well understood. However, alternative mechanisms whereby antibodies might possibly interfere with the binding properties of bacteria and thereby prevent bacterial colonization, apart from specifically blocking the adhesin-receptor interaction, remain less clear.

The present work was aimed at determining whether specific antibodies raised against the fimbrial shaft subunits may influence its mechanical properties, i.e., elasticity and kinetics. We assessed force-extension curves of P-fimbriae by unwinding the shaft in the absence and the presence of polyclonal anti-PapA antibodies using force measuring optical tweezers with sub-pico-Newton (pN) force resolution. In the presence of antibodies, our data showed a significant change in the unwinding force and the shape of the force curves, clearly demonstrating the altered bio-mechanical properties of P-fimbriae. We posit that antibodies, in addition to their major role in marking bacteria as ‘foreign’, may also interact with fimbriae in a way that can directly affect their ability to withstand shearing forces.

## Results

### Immunofluorescence and transmission electron microscopy of bacteria expressing P-fimbriae and labeled with anti-PapA antibodies

**Figure 1:**
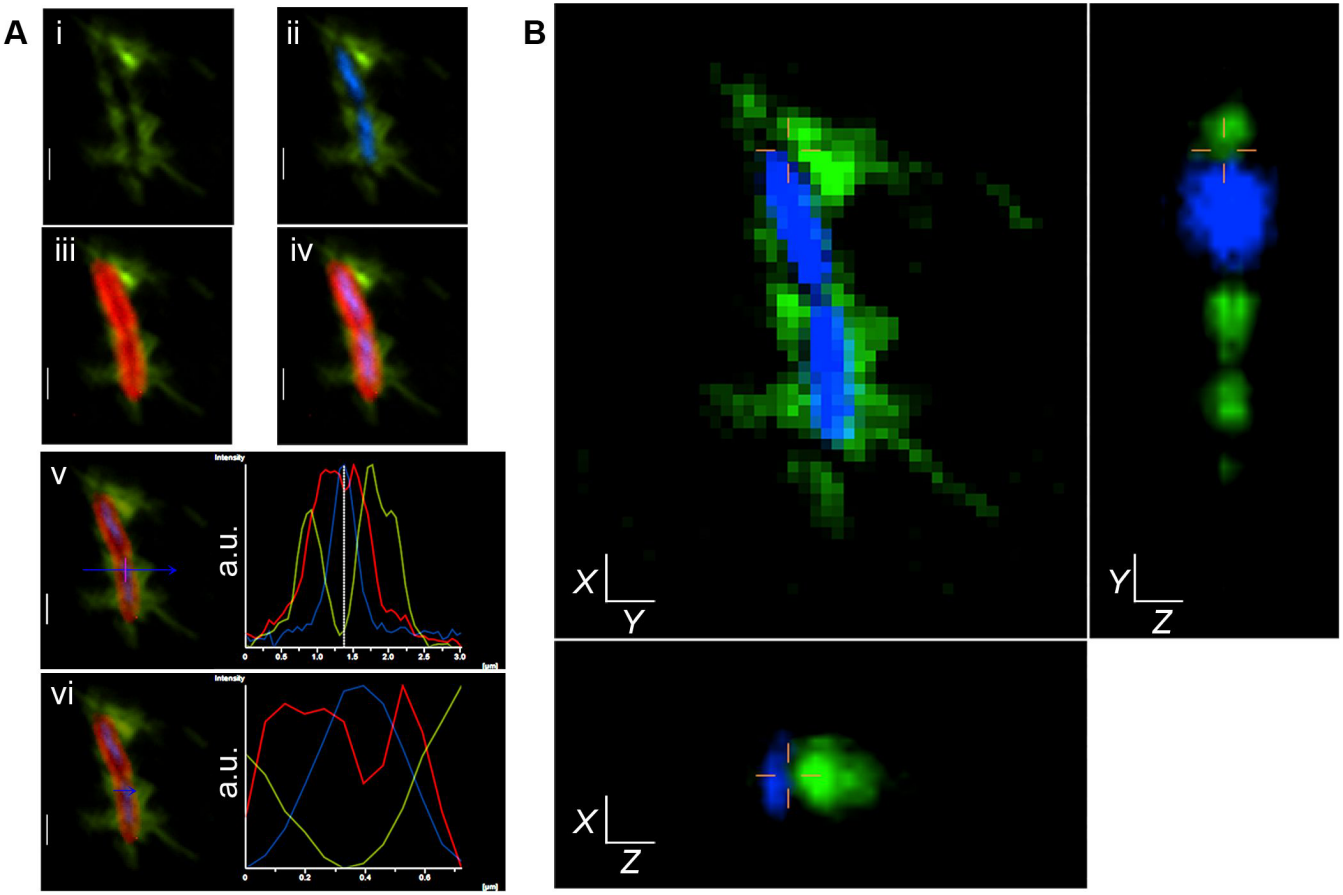
Localization of P-fimbriae at the cell periphery using anti-PapA antibodies. (A) Epi-fluorescence micrograph showing - Alexa Fluor 488 (green) binding to anti-PapA antibodies linked to PapA subunits (i), its co-localization with DAPI (blue) stained nucleoid (ii), with FM4-64FX (red) labeled cell membrane (iii), and with both (iv). Fluorescence intensity curves for Alexa Fluor 488 (green), DAPI (blue), and FM4-64FX (red) at a randomly selected location, shown with the blue arrow, shows minima for green curve coinciding with the maximum of blue curve, marked with the vertical lines and vice versa (v), with an intersecting red curve (vi). Y-axis of the curve represent fluorescence intensity in arbitrary units (a.u.) and X-axis shows distance across the blue line in μm. (B) 3D rendering of same cells using confocal microscopy verifying peripheral localization of Alexa Fluor 488 (green) compared with cell interior occupied by nucleoid (blue). Site of scan is indicated by cross lines. The scale bar is 1 μm.

A typical example of epi-fluorescence and confocal images of bacteria in the presence of high (0.2 μg/ml) and low (2.2 ng/ml) concentrations of anti-PapA antibodies are shown in Fig. 1 and Fig. S1, with control images shown in supplementary Fig. S2. Localization of anti-PapA antibodies bound to cells was detected by Alexa Flour 488 conjugated secondary antibodies (AF488), and it was evident at the cell exterior as a peripheral spray, confirming the peritrichous positioning of P-fimbriae (green; Fig. 1A-i). The pattern of AF488 localization was consistent even when a 100 fold lower concentration of anti-PapA antibodies was used (supplementary Fig. S1). To distinguish between internal and external localization, we marked cell interiors by DNA binding stain DAPI (blue, Fig. 1A-ii) and cell boundaries using membrane dye FM4-64FX (red, Fig. 1A-iii). Co-localization of AF488 with both cell membrane (red) and DNA (blue) clearly shows the differential spatial occupancy of anti-PapA antibodies around the cell periphery (Fig. 1A-iv); a fluorescence intensity profile plotted across a randomly selected zone, marked with an arrow, clearly shows a decline in AF488 intensity in the cell interior when compared with the cell exterior (Fig. 1A v, vi).

To verify the peritrichous localization of anti-PapA antibodies, we recorded fluorescent signals from the same cells at different focal depths using confocal settings. Three dimensional (3D) rendering of the confocal images clearly indicated the presence of anti-PapA antibodies at the cell exterior without overlapping with the nucleoid (Fig. 1B, supplementary movie S1). The secondary antibody, Alexa Fluor 488, on its own did not show any non-specific localization in the absence of primary antibody (Fig. S2A). To further verify the specificity of our anti-PapA antibody-fimbrial interaction, we performed similar analysis using antibodies raised against outer membrane protein A (OmpA). A peripheral spray of fluorescence was not observed when anti-OmpA (Fig. S2B) antibodies were used as the primary antibodies. Immunofluorescence (IF) images clearly showed localization of anti-PapA antibodies that differed from that of anti-OmpA antibodies.

We confirmed the interaction of anti-PapA antibodies to its specific target, PapA proteins, using western blot (WB) analysis. A WB of total cell lysate using anti-PapA antibodies showed an ~17 kDa band only in the case of HB101/pHMG93 cells. This band was absent in the standard laboratory strain, MG1655, which lacks the *pap* gene cluster (Fig. S3). Similarly, WB data on total cell lysates using anti-OmpA antibodies showed the presence of specific bands at ~37 kDa, consistent with the size of OmpA protein (Fig. S3). The band representing OmpA was present in both cell lysates, as expected since OmpA is present in both bacterial strains. We further confirmed the specific binding of anti-PapA antibodies to fimbriae by transmission electron microscopy (TEM) imaging, see Fig. S4.

A low concentration of anti-PapA antibodies was chosen for our experiments to minimize cross-linking of adjacent fimbriae by two arms of a single antibody molecule. The low number of antibodies cross-linking two fimbriae and the peripheral localization of the anti-PapA antibodies was confirmed directly by TEM imaging. However, as expected, since PapA subunits on two fimbriae are identical, the data indicate that some cross-linking did occur. From TEM images, see Fig. S5, we assessed the average length of fimbriae to be ~0.74 ± 0.33 μm and with the known rise per PapA subunit (7.54 Å) the number of subunits per fimbriae could be estimated. By counting the number of gold particles in the TEM images we estimated the number of gold particles per subunit to 0.02 (*n* = 82).

### Force spectroscopy experiments of P-fimbriae from HB101/pHMG93 strain

**Figure 2:**
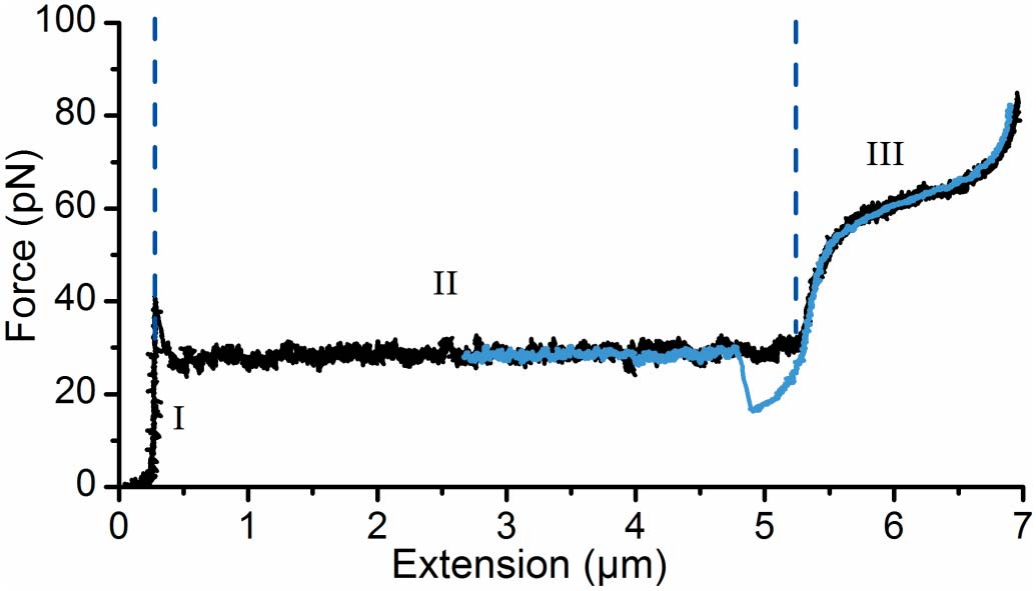
Force spectroscopy experiments in absence of antisera. Typical unwinding (black) and rewinding (blue) curves of a P-fimbria (expressed by *E. coli* carrying the plasmid clone pHMG93) as assessed with force measuring optical tweezers under steady-state conditions in PBS buffer. The response can be divided into three distinct regions, (I) stretching of the quaternary structure, (II) unwinding of the shaft (the distance between the two vertical dashed blue lines), (III) stretching of the head-to-tail bonds.

The *pap* operon in plasmid pHMG93 is transcribed from a constitutively active promoter resulting in more fimbriated bacteria than in a previously described system using the pPAP5 plasmid^23^,^24^,^25^. To verify that the force-extension responses of P-fimbriae expressed by HB101/pHMG93 were identical to that of P-fimbriae present on strain HB101/pPAP5, we performed experiments as described in^23^. A typical force-extension response for an extension velocity of 0.1 μm/s, i.e., under steady-state conditions, is shown in Fig. 2. In the curve the force initially increases linearly with extension (labeled ‘I’), representing an elastic stretch of the fimbria shaft. When the force reaches ~28 pN a transition to a constant plateau force (labeled ‘II’) indicates sequential unwinding of the shaft, as described earlier^23^. The length of this unwinding region is marked by the two vertical dashed lines. In some experiments, as is also evident from the representative data shown, an additional force higher than 28 pN was needed to start unwinding of the fimbria and it appeared as if some extra force was required to break the initial bond. However, we have previously shown that this higher initial force originates from the shaft's attachment to the bead and not from the actual unwinding of fimbriae and therefore this does not affect the interpretation or outcome of the measurements of present experiments^23^. At the end of the plateau the force once again increases, this time non-linearly (labeled ‘III’), representing a stretch and random phase transition of the head-to-tail bonds. Random bond opening gives rise to the slightly tilted force increase. This force-extension response is identical to previous experiments performed on P-fimbriae encoded from the pPAP5 and pPAN5 plasmids^25^.

The blue curve represents the rewinding phase, i.e., movement of the stage is reversed and the tension on the fimbrial shaft is gradually released allowing it to rewind. For this particular experiment the reversed movement of the stage was stopped at 2.6 μm. As is seen, the two curves are overlapping showing that the experiments are performed at steady-state^23^. However, at 5 μm the two curves do not overlap, the force in the rewinding phase drops down to 15 pN before a sharp transition back to the constant force plateau^26^. This particular behavior is a consequence of the helical shape of the shaft and how adjacent layers are connected. For a P-fimbrial shaft the *n*^th^ subunit in the helical structure is connected to the *n* + 3^rd^, thus when the fimbria has been unwound a certain amount of slack is needed before the two subunits are close enough to form a layer-to-layer (LL) interaction^27^. Please note that LL is denoted “turn-to-turn” in some references. An additional force spectroscopy measurement showing the consecutive unwinding and rewinding of a P-fimbria in the absence of antibodies is presented in Fig. S6. As is seen, the force response is constant between the extension cycles.

### Impact of anti-P-fimbriae antibodies on the mechanical properties of P-fimbriae

**Figure 3:**
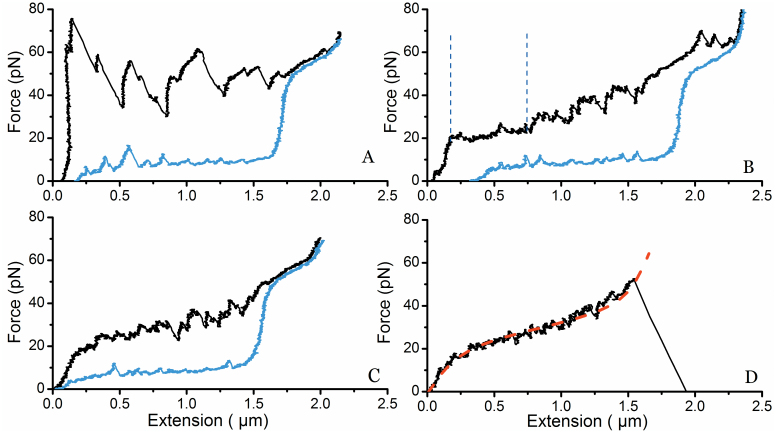
Force spectroscopy experiments in presence of antisera. Sequence of four consecutive force measurements of a P-fimbria unwinding-rewinding being influence by anti-PapA antisera. (A) In the first experiment several force peaks occur when unwinding (black curve) the structure. These peaks correspond to the detachment of a Fab arm of the IgGs. The rewinding curve (blue curve) does not show the formation of a distinct nucleation kernel, which generally would have been seen at 1.5 μm, however, small peaks are found indicating that some (but not all) subunits of the shaft form helical regions. Thus the shaft does not retain all of its helical form. (B) The same fimbria is again extended and a force plateau is initially seen, representing unwinding of the helical regions in the previous experiment. Again, the force rises non-linearly during the extension. The rewinding curve shows fewer force peaks than were observed in the previous rewinding now indicating that fewer helical regions are formed. (C) No distinct force plateau can be seen indicating that the subunits in the helical shaft now open stochastically. (D) Unwinding of the fimbria does not show any sign of sequential opening of subunits indicating that the fimbria no longer has any helical regions available to unwind. The two-state Sticky-Chain model for individual bond-opening was fitted to the data, and is shown as a red dashed line.

Next, we performed steady-state force spectroscopy experiments on fimbriae (*n* = 60) in the presence of antibodies with the same concentrations as used in the IF experiments, i.e., 0.2 μg/ml. At this concentration we expected a low number of cross-linked fimbriae and that the force-extension response would most likely originate from one fimbria. A typical experiment with four consecutive unwinding and rewinding responses is shown in Fig. 3; three additional unwinding and rewinding curves from other fimbriae (in the presence of antibodies) are presented in Fig. S7. In the first extension phase (black curve) several force peaks are present. Compare the force response with Fig. 2. These are believed to originate from either the clamping of several layers of the helical shaft preventing the sequential unwinding at the plateau force or possible cross-linking with other fimbriae. At higher force we expect that one arm of the antibody detaches from the fimbria and unwinding occurs with a sharp drop in force. As is seen in Fig. 3A the force peaks at high forces >50 pN are similar to that of the first part of region III of a fimbria in the absence of antisera, thus indicating that a region between two or more antibodies on the fimbria has unwound and the tensile force is stretching the head-to-tail bonds, see Fig. 2 and reference^23^.

During rewinding (blue curve) the fimbria tries to regain its original helical form. However, the force curve does not show a nucleation kernel with a return to a force that follows the unwinding curve (~28 pN) as is seen at ~5 μm in Fig. 2 (fimbria in the absence of antisera). Instead the force drops ~10 pN and continues slowly decreasing (almost horizontally) with rewinding. However, a few force peaks can be seen in the rewinding process indicating that the fimbria is trying to regain its helical form. Figure 3B shows the second measurement on the same fimbrial shaft. During the unwinding phase it can be seen in the beginning of the curve that a short section is sequentially unwound (constant force plateau) and that there are fewer force peaks. The force is also slightly increasing with further extension, consistent with a combination of sequential unwinding and random opening and closing of the LL bonds. In the second round of rewinding the curve has even fewer force peaks than seen in the previous rewinding of the fimbria. In the third measurement the force increases continuously with extension showing that the fimbria now appears to unwind randomly (Fig. 3C). This implies that there are only a few LL interactions that can unwind sequentially. The rewinding curve is now almost smooth with only two small peaks.

Eventually, as shown in Fig. 3D, the force-extension curve increases non-linearly, similar to that of the third region (III) of a fimbrial shaft subjected to force in the absence of antisera, before it detaches from the bead. The non-linear increase in force at all extension lengths indicates that the structure now opens only stochastically, without any sequential unwinding. Therefore, we anticipated that the sticky-chain model for individual bond opening would suitably fit the force-extension response of the 4^th^ force-extension curve. The sticky-chain model^23^ was fitted to the data (red dashed line), and it excellently reproduces the force-extension curve^28^. The model parameters for the best fit are given in the supplementary material.

We also performed force spectroscopy experiments with 100-fold lower concentration of antibodies, i.e., at 2.2 ng/ml. Three typical curves from these experiments are shown in Fig. S8. Force-extension curves at this low concentration of antibodies produced fewer and smaller peaks as compared with high concentrations. Our control measurements with anti-OmpA antisera produced force curves similar to that shown by fimbriae in the absence of any antisera, see Fig. S9.

To further investigate if anti-PapA antibodies locked consecutive layers via bivalent binding we made additional studies after cleaving IgGs into Fab fragments. Preparation of the Fab fragments and the purification procedure are given in Supplementary materials, and the corresponding SDS-PAGE gel is shown in Fig. S10. Force spectroscopy experiments on individual fimbriae were thereafter performed in the presence of Fab fragments. A representative experiment of a consecutive force measurement of a P-fimbria in the presence of 0.2 μg/ml Fab fragments is shown in Fig. S11. The total number of measurements was *n* = 42. In comparison to the force curves shown in Fig. S6 (only buffer) and Fig. 3 (with IgGs) the curves obtained in presence of Fabs show that normal unwinding and extension could occur whereas the rewinding phase is influenced by a high degree of mis-foldings indicating that Fab fragments impair rewinding mechanics.

### Extension length of P-fimbria with and without anti-PapA antibodies

**Figure 4:**
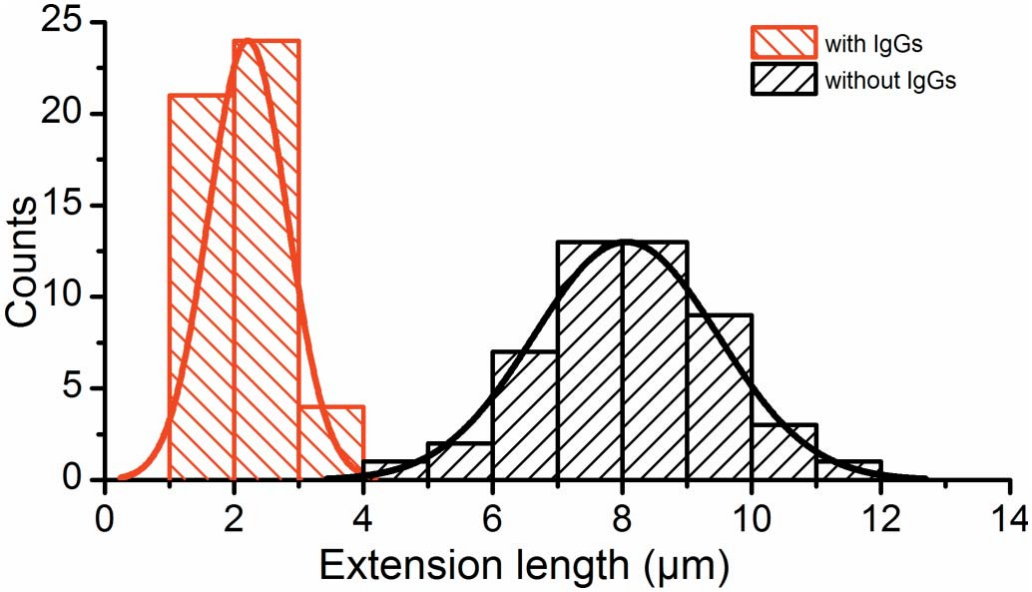
Extension lengths of fimbriae in the presence and absence of antibodies. The histogram to the left side represents the extension lengths of fimbriae in the presence of antibodies with a mean extension length of 2.2 ± 0.6 μm, (*n* = 50). The histogram to the right represents data obtained in the absence of antibodies with a mean extension length of 8.0 ± 1.4 μm, (*n* = 50).

The unwinding length of the shaft under a constant force, which is shown as the distance between the two vertical blue dashed lines in Fig. 2, in the presence of antibodies was significantly shorter, see Fig. 3B. Figure 4 shows the distribution of the unwound lengths of P-fimbriae without and with antibodies (*n* = 50 for each histogram). The histogram on the left (red) represents the extension length distribution of P-fimbriae in the presence of anti-PapA antibodies with a mean value of 2.2 ± 0.6 μm. The histogram on the right shows the distribution of P-fimbriae (black) in the absence of anti-PapA antibodies with a mean value of 8.0 ± 1.4 μm. Our data thus strongly indicate an extension length significantly shorter in the presence of antibodies, i.e., significantly reduced elasticity of the fimbriae. Extension length was unaltered in presence of Fab fractions alone (Fig. S11).

### Characterization of the multiple force peaks created in the presence of anti-PapA antibodies

**Figure 5:**
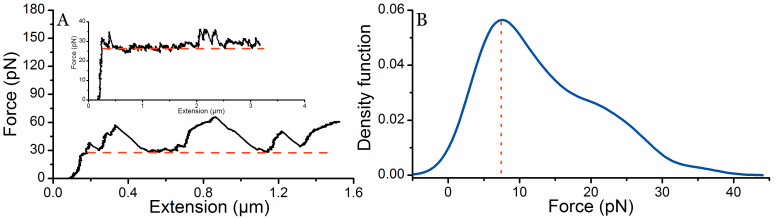
Detachment force of antibodies. Panel A shows the force-extension curve of a P-fimbria in the presence of anti-PapA antibodies. The force peaks (the parts of the force curve above the level of the unwinding force indicated with a red dashed line) represent the rise attributed to the force needed to disrupt an antibody-PapA interaction. The subsequent drop represents shaft unwinding after this disruption, which leads either to a plateau of continued unwinding or another rise in force due to another antibody-PapA interaction. The inset shows a detailed unwinding curve with small force peaks appearing above the level of the unwinding force. Panel B shows a kernel density estimate curve (bandwidth of 3.1) of the detachment forces during the unwinding process with a peak at 7.5 pN (*n* = 120).

In the presence of anti-PapA antibodies the force-extension curves significantly differed from normal curves obtained in the absence of antibodies and, as described above, multiple force peaks appeared during unwinding. Figure 5A shows a force-extension curve of a P-fimbrial shaft in the presence of anti-PapA antibodies indicating high force peaks whereas the inset shows an example of a curve with lower force peaks. Nevertheless, these force curves are clearly different from the typical force curves obtained in the absence of antibodies (Fig. 2). We assessed the height of the force peaks (for concentrations of 2.2 ng/ml and 0.2 μg/ml) within an interval of >3 to <35 pN above the normal unwinding force of 28 pN. These peaks thereby represent the additional force needed to change the orientation of a subunit from a perpendicular to parallel direction relative the tensional force vector. Figure 5B represents a kernel density estimate curve of these detachment forces during the unwinding process for steady-state conditions. The distribution curve yields a peak at 7.5 pN (*n* = 120).

### Conceptual modeling of anti-P-fimbriae antibody interactions on P-fimbriae

**Figure 6:**
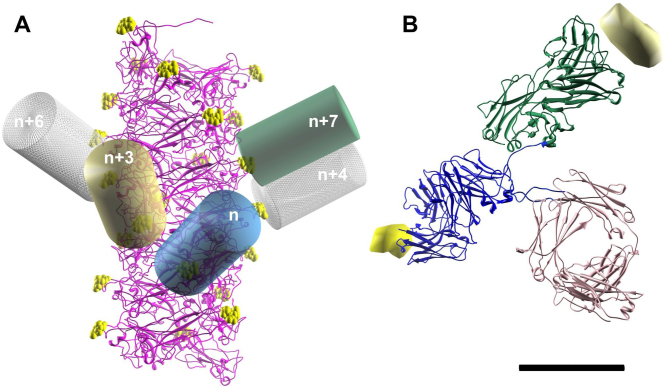
Conceptual modeling of anti-P-fimbriae antibody interactions on P-fimbriae. (A) IgGs modeled as low resolution cylinders and fitted to Val 11 in the P-fimbriae helical reconstruction map^29^. (B) An epitope is shown at 2 nm resolution when bound to a Fab-antigen (from pdb 3HFM) shown as a ribbon diagram. The scale bar is 5 nm.

In order to estimate both the maximum number of antibodies interacting with a single P-fimbrial shaft as well as the number of layers being clamped by an antibody, we used the P-fimbriae helical reconstruction map (Fig. 6A^29^;) and fitted a representative antibody class – immunoglobulin G (IgG) model (pdb 1IGT; Fig. 6B) to a potential epitope on the shaft fimbrial subunits. An epitope shown at 2 nm resolution is included in Fig. 6B, bound to a Fab-paratope (from pdb 3HFM). One arm of the IgG was attached to the *n*^th^ subunit of a fimbria and denoted the primary arm. We have chosen PapA Val 11 as our representative epitope for IgG binding, with the atoms of Val 11 represented by yellow spheres in Fig. 6A. A cylinder of diameter 5 nm and length 10 nm provides a good low resolution visualization of one IgG arm, and the primary arm is shown as a blue cylinder in Fig. 6A.

We thereafter applied a set of three constraints to find additional subunits to which the second arm of the IgG could attach. First, since the two arms of an IgG are allowed to be separated by a distance of ~10 nm via a flexible hinge joint, the secondary arm was constrained to only attach to the shaft within a spherical volume 10 nm in diameter; see supplementary movie S2. Second, since the shaft is constructed of identical PapA subunits and since the two arms of the IgGs have identical antigen-binding sites, we only allowed the IgG to attach to Val 11 residues along the filament. In addition, the secondary arm had to attach at the same angle relative the shaft as the primary arm. Third, steric clashes of the two arms were taken into account requiring that attachment of the arms must be at least 5 nm apart.

By using these constraints we looked at the possibility of the second arm binding to neighboring subunits (relative to the *n*^th^ subunit). The plausibility of potential interactions is given in Table 1. We found that the highest likelihood for an anti-PapA antibody to bind would be between the *n*^th^ and *n +* 7^th^ subunits. This is then illustrated by the primary arm (blue cylinder) and secondary arm (green cylinder) in Fig. 6A. It is also plausible, even though the arms are close, that attachment could take place on the *n*^th^ and *n +* 3^rd^ (tan cylinder) subunits. Our modeling thereby implies that a maximum ratio of 1:4 (allowing the *n*^th^, *n* + 7^th^) or 1:2 (allowing the *n*^th^, *n* + 3^rd^ and *n* + 7^th^) antibodies per subunits can attach to the shaft.

## Discussion

It is well established that fimbriae are virulence factors that are essential for the attachment of UPEC to their host cells. An absence of fimbriae or presence of non-functional fimbriae reduces bacterial infectivity^30^,^31^. P-fimbriae of UPEC strains are well studied for their role in establishment of clinical diseases, including their mechanical property that provides resilience to the various forces imposed by the host^32^,^33^,^34^. In this study, we investigated the mechanical properties of these fimbriae under continuous force and in the presence of antibodies that specifically bound to the fimbrial shaft. Our data showed that in the presence of anti-PapA antibodies the force response of P-fimbriae was significantly altered in comparison to experiments without antibodies and what has previously been reported. We noticed that the regions that could be unwound at a constant force decreased for each cycle until the force response resembled that of stochastic bond opening indicating that antibodies blocked proper rewinding. In some experiments the results indicated that binding of multiple antibodies at specific regions clamped layers shut that prevented further unwinding without the addition of a very large force. Thus, binding of shaft-specific antibodies significantly reduced the elasticity of fimbriae. Rewinding may be compromised because unwound fimbriae, most likely, cannot bend sufficiently in the presence of antibodies to provide a nucleating site, which is needed to initiate rewinding into a helical filament^26^.

From our model and the known length of a subunit we predict that the second arm of the antibody bound to fimbrial subunit *n* most likely binds to fimbrial subunits *n* + 3 or *n* + 7 of the intact helical filament, and *n* + 2 or *n* + 3 of the unwound structure. Another possible outcome is that the second arm of an antibody binds to a subunit far from its neighbor by virtue of long loops created by unwinding. This would clamp the ends of the long loop, preventing productive rewinding of subunits within the loop. By using the conceptual model the ratios of antibodies per subunits can be calculated to 1:4 or 1:2. This implies that for a μm long fimbriae at most ~325 or ~650 antibodies can bind, which is substantially more than found in the TEM images at the concentration of 4 ng/ml, where ~20 antibodies per fimbriae were seen implying a ratio of 1:50. Our calculations are based on the proposed structural arrangements of the interacting partners – fimbria and intact IgGs, and not on the actual affinity of antigen-antibody interaction. In force spectroscopy experiments run at the lowest concentration of 2.2 ng/ml, only a few force peaks (~10) were seen indicating that the shaft was marginally decorated. At the 100-fold higher concentration, 220 ng/ml, the force-extension responses were significantly altered indicating that the shafts were widely decorated, which is reasonable since at this concentration we expected the fimbriae to be more or less fully decorated.

A P-fimbria unwinds at a force of 28 pN. For multi-fimbriae unwinding, i.e., when several fimbriae are attached to the probe bead and extended, the unwinding force is a multiple of 28 pN. This is shown in Fig. S12 (upper panel) where initially four fimbriae were attached. Upon extension these fimbriae detached sequentially as seen by the sharp force transitions. The force response of two fimbriae cross-linked by an antibody would give rise to a response similar to the one seen in lower panel of Fig. S12, since the applied force would be shared equally by the two fimbriae, or one fimbria would be slack and it would not significantly increase the pulling force. In addition, if one fimbria is unwound and cross-linked to an adjacent fimbria in its helical configuration a significant force >100 pN would be required to initiate unwinding since a layer in the fimbria must first be opened^23^. In this work, the force response in the presence of antibodies does not resemble that of multi-fimbriae force data. Most likely the altered force-extension response originates from intra-shaft attachment by antibodies that cause a requirement of additional force for fimbria unwinding.

Other work aimed at studying the physical properties of IgGs attachment indicates a slip bond behavior, and that a force of ~20–150 pN is required for breaking the antibody-antigen bond^35^,^36^. In our setup tensile force was applied to the fimbria and most likely the antibodies were attached perpendicular to the fimbrial axis. Thus, the angular position of the ligand (the antibody epitope on the subunit) is continuously altered under force, and the antibody is attached to this rolling target. We expect that the timescale of our experiments with slowly increasing force, and rotation of the ligand in the optical trap during the experiments results in the lower forces measured for detachment (~7.5 pN). In addition, since our experiments were performed under steady-state conditions we can conclude that the higher unwinding force measured in some of our experiments is attributed to the effect of antibodies on the fimbriae and not due to dynamic effects that are known to cause increased forces for unwinding at higher velocity^37^.

On average, the unwinding length of fimbriae in the presence of antibodies was significantly shorter than for fimbriae in the absence of antibodies, even when measuring the first unwinding cycle (Fig. 3). Since the antibodies used in these experiments are polyclonal, their attachment is not limited to a single epitope. Therefore, it is highly plausible that multiple antibodies are able to bind such that they “lock” regions of a fimbria. An extremely high force would then be required for breaking the layer-to-layer interaction, so that the fimbria would either not unwind due to limits in the force range of the apparatus or would detach from the bead before the unwinding force is reached.

From the force peak data it is possible to approximate the number of layers being clamped by antibodies. We assessed the force peak above the normal unwinding force for all data curves. The density function of the force peak data shows a peak around 7.5 pN, which is close to the value of 4–6 pN derived using our conceptual model that an antibody can bind the *n*^th^ and *n* + 7^th^ subunits (see Supplementary materials for calculations). However, the wide distribution of peak forces indicates that the fimbriae in some experiments also are extensively decorated by antibodies resulting in high forces of 20–30 pN needed to unwind layers. In addition, the high force drops together with a sudden fimbriae elongation of 0.1–0.2 μm suggest that unwinding occurs in spurts at high IgG concentrations. It is likely that a region of a fimbria is decorated with IgGs and when the force is high enough to break an IgG bond an adjacent IgG will be exposed to a momentarily increased force that overwhelms its binding. We hypothesize that this may create a cascade effect that rips the decorated region apart.

Bivalent binding of antibodies to a fimbria was investigated by cleaving IgGs into Fab fragments. The data showed that no force peaks appeared during unwinding, which otherwise would be expected if Fabs were able to bind to and crosslink two consecutive layers. The force response in the presence of Fab fragments was thereby significantly different from that observed when intact IgGs were used. However, during rewinding of the fimbria a high degree of mis-foldings was present indicating that Fab fragments can bind shaft subunits and impair proper rewinding of the helical shaft. This response is similar to what was found by Klinth et al. who investigated if the PapD chaperone could change the biomechanical compliance of P-fimbriae^9^. They showed that PapD only bound to unwound fimbriae, which is expected since the recognition site of a PapA subunit is hidden when the shaft is in its helical state, and that the rewinding process was impaired by PapD. Thus, both Fab fragments and PapD require unwinding of a fimbria to change its biomechanical compliance whereas IgGs directly impair the biomechanical compliance by locking layers in the fimbriae and making it relatively stiffer.

The sticky-chain model for stochastic opening of bonds was fitted to the data presented in Fig. 3D. The good fit of the model and the reduced length of the force plateau seen in consecutive experiments with the same fimbrial organelle suggest that antibodies prevent proper LL interactions to form when rewinding is allowed. Unwinding therefore takes place stochastically; any subunit in a layer has the possibility to switch conformation during the timescale of the experiment, giving rise to the pseudo elastic response similar to that of region III in Fig. 2 when unwinding fimbriae in the absence of antisera.

Data from WB, IF, and TEM validate the specificity of our force spectroscopy results, supporting a mechanism in which unwinding and rewinding of P-fimbriae in the presence of anti-PapA antibodies is due to specific interactions between antibodies and fimbriae. To estimate the number of layers that could be clamped by a single antibody, an atomic model structure of an IgG was fitted by a set of constraints to the P-fimbriae helical reconstruction map (using Chimera software). We attached one of the Fab arms to an epitope and looked for how far the other arm could reach without breaking the constraints. This is not a quantitative method but it gives a reasonable estimate of the number of subunits that can fit between the two attachment points. In addition, we showed by a similar approach that it is likely that an antibody should be able to bind two subunits on an unwound fimbrial polymer thus making it possible to create loops. We believe that these loops can be substantial since it is known that a large amount of slack is needed for nucleation of a stretched fimbria, i.e., numerous subunits are involved in making a nucleation kernel.

**Figure 7:**
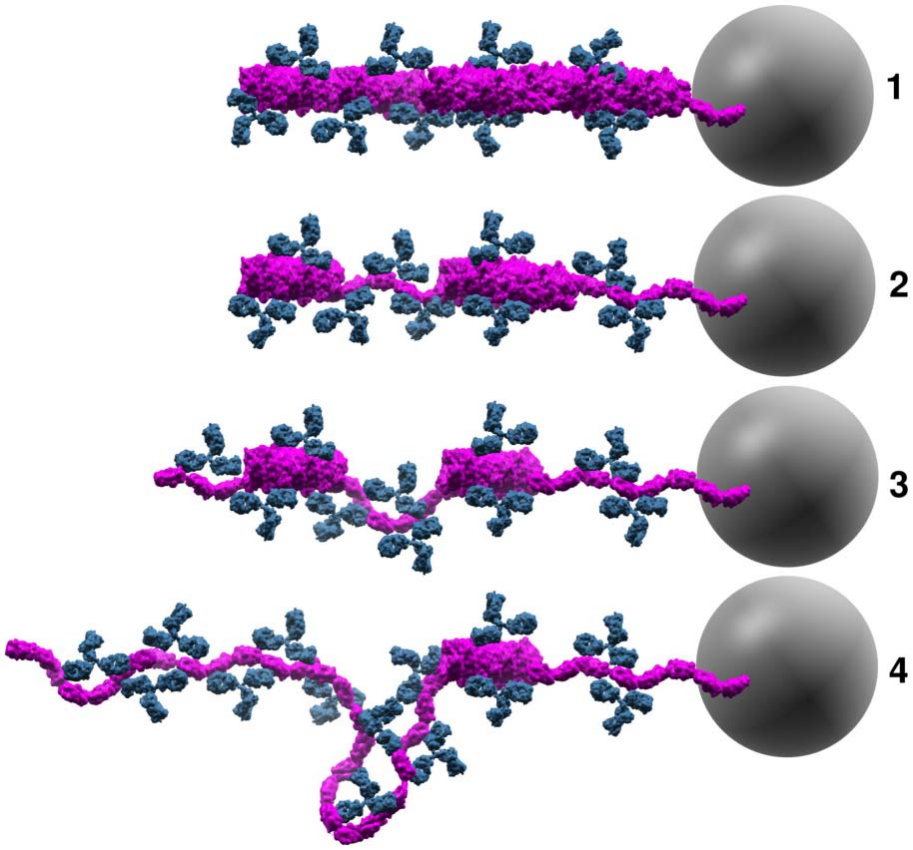
Conceptual model of antibodies attaching to P-fimbriae. A schematic representation of our conceptual model for molecular events monitored in the force spectroscopy experiments shown in Fig. 3. The panels represent the configuration of the fimbria (magenta) and antibodies (blue) prior to, and during, the extension phase. Antibodies initially interacted with the helical shaft. After the fimbria was unwound some antibodies prevented proper rewinding by interfering with the LL interactions. Loops were able to form when antibodies bound two subunits in an unwound configuration. The gray sphere illustrates the trapped 2.5 μm bead (not drawn to scale) that was used as probe in the force spectroscopy experiments.

Our experiments were conducted using polyclonal PapA antibodies that provide the possibility of binding both to the helical shaft and cryptic epitopes that might be exposed only upon unwinding of the fimbriae. Considering how the experiments were conducted and the results obtained we propose the following conceptual picture, as seen in Fig. 7, prior to each extension of the experiment shown in Fig. 3. 1) Initially, antibodies were only attached on the surface of the fimbriae since the strong interactions between layers do not allow for spontaneous unwinding; 2) after the first unwinding/rewinding cycle some layers opened and were held open by the antibodies, thus preventing proper orientation of the subunits back into their helical geometry. However, some regions of the shaft had several antibodies attached and the unwinding force was not sufficient to open these layers; 3) subsequently a few more layers were opened and prevented from properly rewinding; 4) eventually no intact helical regions could be unwound. Layers could open stochastically giving rise to the pseudo elastic force-extension curve that is similar to random opening of the head-to-tail bond (c.f. region III in Fig. 2).

The data presented here show that polyclonal anti-PapA antibodies interact and considerably reduce the elastic properties of the P-fimbriae, which are helix-like adhesion organelles. For other helix-like organelles similar to P-fimbriae, e.g., the type 1 fimbriae and the CFA/I fimbriae, it has been shown that the elasticity of the shaft is important for sustained adhesion^2^,^5^,^7^. Our observations therefore raise a question: could the attachment of antibodies to fimbrial shaft be an active mechanism that obstructs sustained adhesion of bacteria to host cells?

We have presented a study that investigates the direct influence of antibodies at a single organelle level on fimbriated cells. Polyclonal antibodies were used in this study to maximize the response; however, future studies will aim to investigate the effect of antibodies targeted against specific fimbrial subunit epitopes and by the use of e.g. flow-chamber experiments it should be possible to quantify the differences under fluid drag. Our findings provide new information that antibodies that specifically bind to fimbrial shafts can strongly interfere with their unwinding and rewinding mechanics. These observations suggest a new possible biological role of secretory antibodies in combating bacterial infections by directly interfering with the mechanical properties of virulence factors, especially surface adhesins. Our observations should therefore be considered in design of antibody-based therapeutics.

## Methods

### Bacterial strains and antisera

The *E. coli* strain HB101 was used as a host strain for the plasmid pHMG93 expressing P-fimbriae^38^. The *E. coli* K-12 reference strain MG1655^39^ was used as a negative control. Bacteria were cultured on trypticase soy agar (TSA; Becton, Dickinson and Company, NJ USA) at 37°C for 24 h.

Polyclonal antibodies against PapA subunits of P-fimbriae were raised in rabbit. The specificity of antibody binding to PapA subunits was assessed using western blot analysis, and immunofluorescence microscopy. Polyclonal anti-OmpA rabbit antisera were used as control antibodies^40^.

### Immunofluorescence (IF; Epi-fluorescence and confocal) and Transmission electron microscopy

For imaging, an isolated colony from the agar plate was harvested and suspended in 500 μl of filtered phosphate buffer solution (PBS; 10 mM phosphate and 130 mM NaCl, pH 7.4, Sigma-Aldrich). The concentration of bacteria suspension was 4 × 10^5^ CFU/ml (determined by viable cell count). In our experiment we incubated ~4000 cells with either 2.2 ng or 22 pg of polyclonal antibodies in a total reaction volume of 10 μl for 10 min, followed by fixation in 4% paraformaldehyde (pH 7.2). For transmission electron microscopy (TEM) imaging the antibody concentration was 4 ng/ml. All incubations were done at room temperature (RT) unless stated otherwise.

Fixed cells with antibodies were spread on a 0.1% poly-L-lysine coated glass coverslip, washed twice with PBS and treated with 2% bovine serum albumin (BSA) for half-an-hour to block any non-specific binding. After blocking, cells were incubated with a secondary antibody Alexa Fluor 488 donkey anti-rabbit IgG ((H + L) cat. no. A-21206, Invitrogen) for 1 h, followed by washing 10 times with PBS. Coverslips containing stained cells were then mounted onto a glass slide using Dako Fluorescent Mounting Media (Dako) to which 0.1 μg/ml of FM4-64FX(N-(3-triethylammoniumpropyl)-4-(6-(4-(diethylammino)phenyl)-hexatrienyl)-pyridiumdibromide; Invitrogen) and 0.5 μg/ml of DAPI (4′, 6-diamidino-2-phenylindole; Sigma) were added to visualize bacterial membranes and DNA, respectively. To check for any non-specific signal from the secondary antibodies alone, a control sample was treated similarly except that the polyclonal P-fimbrial antibodies were not added.

Micrographs were taken using a Nikon Eclipse 90i fluorescence confocal microscope equipped with a Plan AP VC 100×/1.40 oil objective, C-Hg illuminator, and lasers of 408, 488 and 543 nm wavelengths. Confocal images were acquired using a D-DH-E Motorized Digital Imaging Head and imaging software NIS-element EZ-C1 3.90, and epi-fluorescence images were acquired using a Nikon DS-Qi1Mc camera using NIS-Elements AR 3.2 software. NIS-Elements AR 3.2 software was used to analyze both confocal- and epi-fluorescent data. Images were finally compiled in Adobe Photoshop CS5.

TEM micrographs were obtained by resuspending bacteria in 10 mM Tris-HCL with 10 mM MgCl buffer (pH 7.4) and allowing them to adhere to Formvar-coated Cu-grids for 1 min. The grids were then incubated with anti-PapA antiserum (4 ng/ml) for 10 min. Thereafter, they were thoroughly rinsed with buffer and subsequently incubated with 10 nm gold nanoparticles (Biocell GAR10) conjugated with goat anti-rabbit antibodies. The grids were finally rinsed with distilled water and negatively stained with 1% uranyl acetate. Micrographs were taken using a JEOL 1230 transmission electron microscope at 80 KeV and digital images were captured using a Gatan MSC 600CW. The average length, 0.74 ± 0.33 μm, of the fimbriae was assessed via TEM images, see Fig. S5 for a representative TEM micrograph.

### Western blotting

Cultures of strains HB101/pHMG93 and *E. coli* K-12 MG1655 were grown at 37°C on Luria-Bertani plates. Overnight cultures were resuspended in loading buffer containing 62.5 mM Tris-Hydrochloride (pH 6.8), 2% sodium dodecyl sulfate, 5% glycerol, 0.25% β-mercaptoethanol and 0.05% bromophenol blue, and were heated at 97°C for 20 min. Total cell lysates were then subjected to electrophoretic separation on 15% polyacrylamide gels containing 0.1% sodium dodecyl sulfate (SDS-PAGE). Following electrophoretic separation, total cell-proteins were transferred to polyvinylidene difluoride (PVDF) membrane. Individual PVDF membranes were blocked in 5% skim milk prepared in PBS-T (Phosphate buffer saline with 0.1% Tween20), followed by overnight incubation at 4°C with rabbit antisera containing anti-PapA or anti-OmpA antibodies. Membrane blots with primary antisera were washed 3 times, for 15 min each, in PBS-T and incubated with anti-Rabbit IgG linked to Horseradish peroxide (HRP; NA934V, GE Healthcare) for 1 h. Membrane blots were developed with ECL+ detection system (Amersham Biosciences) and detected using Fujifilm LAS4000 system as described elsewhere^41^.

### Force spectroscopy - sample preparation and measurements

Micro-fluidic chambers were made from two cover slides with a single layer of parafilm (100 ± 5 μm thick) with a rectangular shape cut out (length = 10 mm, width = 5 mm) and placed between the cover slides as spacers. Cover slides were mended by heating the flow cell to 80°C for two seconds. In an experiment the chambers were filled completely with the sample and sealed by vacuum grease to prevent evaporation.

Force spectroscopy experiments were thereafter carried out with a custom made optical tweezers (OT) system that has been described in detail elsewhere^23^. Briefly, the OT were constructed around an inverted microscope (Olympus IX71, Olympus, Japan) with a high numerical aperture oil-immersion objective (model: UplanFl 100× N.A. = 1.35; Olympus, Japan). A continuous Nd:YVO_4_ laser (model: Millennia IR) operated at 1064 nm, and run with an output power of ~1 W was used for trapping of 2.5 μm polystyrene beads (Duke Scientific Corp.); these beads were used as handles in the experiments. The position of the beads was monitored by light from a weak HeNe-laser onto a position sensitive detector and prior to each experiment the stiffness of a trapped bead (~140 pN/μm) was calibrated by the power spectrum method^42^. The sample time for calibration was optimized to reduce drift and noise by the method presented in^43^.

The preparation as well as the number of bacterial cells and amount of antisera used in force spectroscopy experiments was similar to what has been described earlier in the IF procedure. To assess a force-extension curve of a bacterium expressing fimbriae, a bacterium from suspension was attached by the laser trap (run at low power not affecting the viability) to a 0.01% poly-L-lysine functionalized 9.7 μm microsphere (Duke Scientific Corp., Palo Alto, CA) that had been immobilized prior to adding sample by heating at 60°C for 1 h on a microscope cover slide. A probe bead was then brought in proximity of the bacterium to attach to a fimbria via non-specific interactions^26^. To attach to a fimbria the bead was moved perpendicular to the bacterium and for each unsuccessful attempt the bead was moved ~100 nm closer until an interaction was formed. With one or several fimbriae attached the bacterium-probe bead was then separated at a velocity of 0.1 μm/s, and the force and position data were sampled at 200 Hz as described in reference^44^.

### Modeling the attachment of antibodies to P-fimbriae using immunoglobulin G (IgG) as representative antibody class

It has been shown that the hinge region of antibody molecules is a flexible domain (Fab) that allows the Fab arms to adopt a wide range of angles and thereby bind epitopes spaced at variable distances apart^45^,^46^. To investigate, by modeling, the binding of two arms of an IgG molecule to helical and unwound P-fimbriae, we input the atomic models of P-fimbriae^29^, PapA dimer (pdb 2UY6), an IgG (pdb 1IGT), and an IgG Fab with bound ligand (pdb 3HFM) into UCSF Chimera software^47^. The model for an unwound PapA fibrillar structure was produced by superposing chains B and C of multiple copies of the PapA dimer, and removing one of the subunits from each copy after the correct alignment was determined.

## Author Contributions

N.M. carried out the force spectroscopy and TEM experiments, B.S. carried out IF, Fab purification and WB experiments. All authors contributed to the planning of the studies and to interpretation of the results. B.S., E.B. and M.A. drafted the main manuscript text and all authors reviewed the final version of the manuscript.

## Supplementary Material

Supplementary Information Movie 1

Supplementary Information Movie 2

Supplementary Information Supplementary materials

## Figures and Tables

**Table 1:** Possible binding positions of two arms from a single IgG

*n*, *n* + 1 not possible; beyond maximum reach
*n*, *n* + 2 not possible; beyond maximum reach
*n*, *n* + 3 maybe steric hindrance
*n*, *n* + 4 unlikely, maximum stretch of tether
*n*, *n* + 5 not possible; beyond maximum reach
*n*, *n* + 6 unlikely, maximum stretch of tether
*n*, *n* + 7 most likely, part of top sticks out
*n*, *n* + 8 not possible; beyond maximum reach
