# Supplementary material for: P-fimbriae in the presence of anti-PapA antibodies: new insight of antibodies action against pathogens
Source: Sci Rep. 2013 Dec 2;3:3393. doi: 10.1038/srep03393 (PMC3848023; doi:10.1038/srep03393)
Supplement: Supplementary Information — Supplementary materials [file srep03393-s3.doc]

# Supplementary material - P-fimbriae in the presence of anti-PapA antibodies: new insight of antibodies action against pathogens

Narges Mortezaei^1#^, Bhupender Singh^2#^, Esther Bullitt^3^, Bernt Eric Uhlin^2^, Magnus Andersson^1^*

^1^Department of Physics, ^2^The Laboratory for Molecular Infection Medicine Sweden (MIMS) and Department of Molecular Biology, Umeå University, SE-901 87 Umeå, Sweden;

^3^Department of Physiology and Biophysics, Boston University School of Medicine, Boston MA 02118-2526, USA

^#^ These authors contributed equally to this work

***Corresponding author:** Magnus Andersson, Department of Physics, Umeå University

SE-901 87 Umeå, Sweden, Tel: + 46 – 90 786 6336, FAX: +46 – 90 786 6673

**E-mail:** [magnus.andersson@physics.umu.se](mailto:magnus.andersson@physics.umu.se)

| 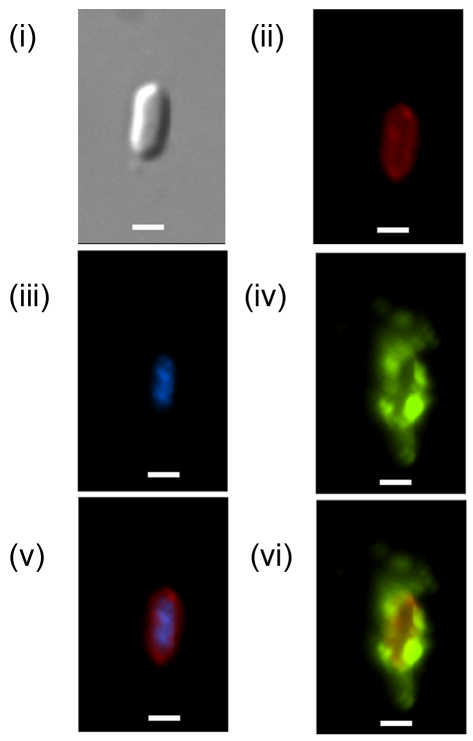 | Figure S1. Localization of P-fimbriae on HB101/pHMG93 cells using anti-PapA antibodies at a concentration of 2.2 ng/ml. Epi-fluorescence micrograph showing (i) bright-field differential interference contrast (DIC) images of selected cells, (ii) FM4-64 labeling of cell membrane, (iii) DAPI stained nucleoid, (iv) Alexa Fluor® 488 binding to anti-PapA antibodies linked to PapA subunits, (v) overlay of FM4-64 (red) and DAPI (blue) signals, and (vi) FM4-64 (red) and Alexa Fluor® 488 (green) signals depicting internal localization of nucleoid and peripheral distribution of anti-PapA antibodies / PapA subunits. The scale bar is bar 1 µm. |
| --- | --- |


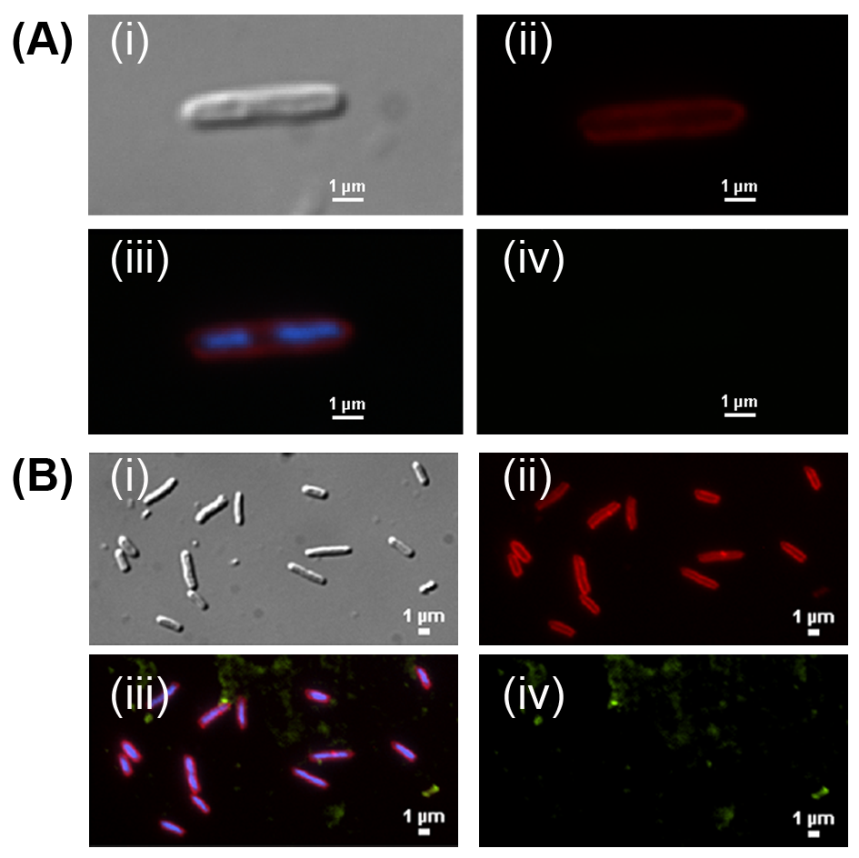


Figure S2. Epi-fluorescence localization of secondary antibody, Alexa Fluor® 488 (green), in HB101/pHMG93 cells: (A) in absence of any primary antisera, and (B) in the presence of anti-OmpA antibodies. Cells were imaged as follows: (i) bright field differential interfering contrast (DIC), (ii) cell-membrane visualization using FM4-64FX (red), (iii) overlay showing cell-membrane (red), nucleoid stained with DAPI (blue) and Alexa Fluor® 488 signal (green), and (iv) the presence of Alexa Fluor® 488 signal alone. (Scale bar = 1 µm)


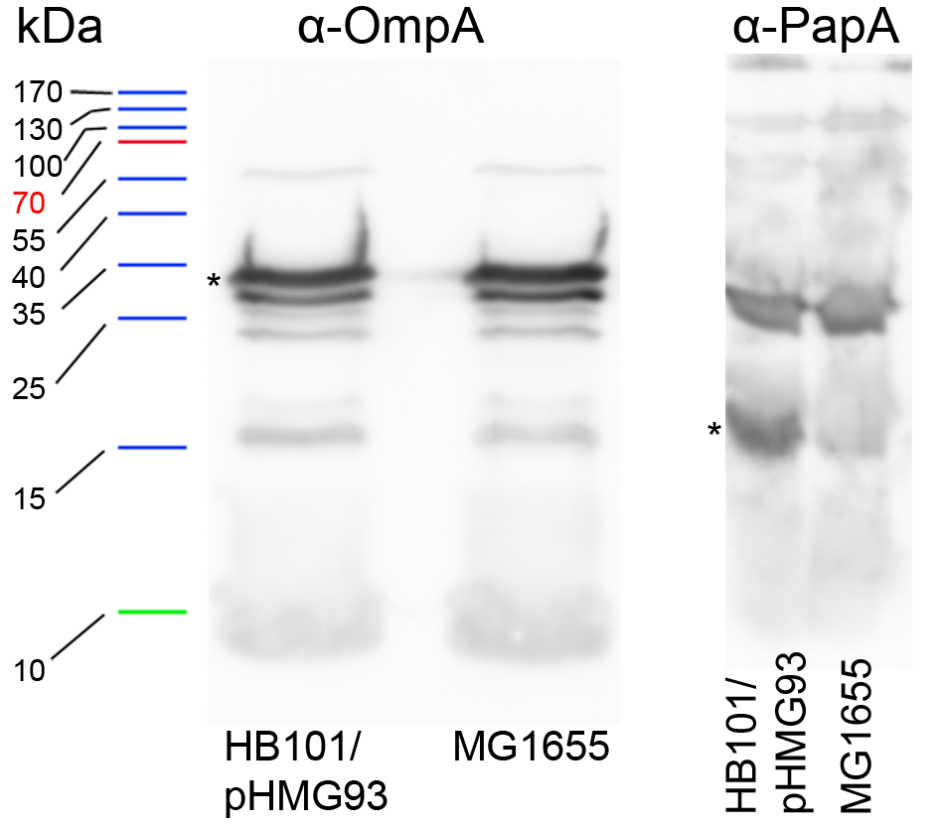


Figure S3. Western blot analysis of total cell lysates from overnight cultures of strains HB101/pHMG93 and MG1655 grown at 37°C, using anti-OmpA, and anti-PapA antibodies. Position of the antibody-specific protein is marked with *.


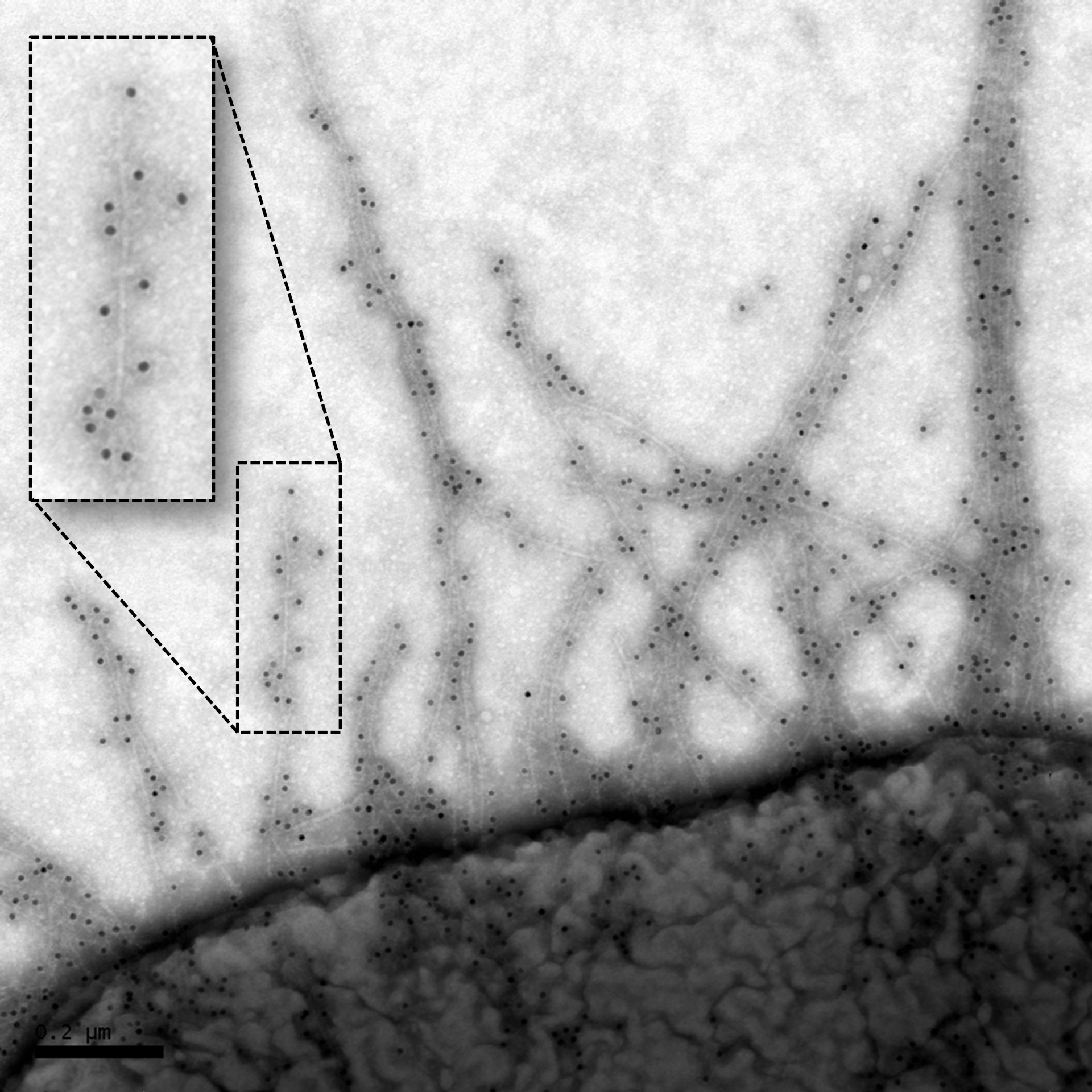


Figure S4. TEM micrograph of a HB101/pHMG93 cell expressing P-fimbriae, labeled with gold particles conjugated with anti-PapA antibodies. The scale bar is 0.2 µm and magnification represents 1.5 × zoom.


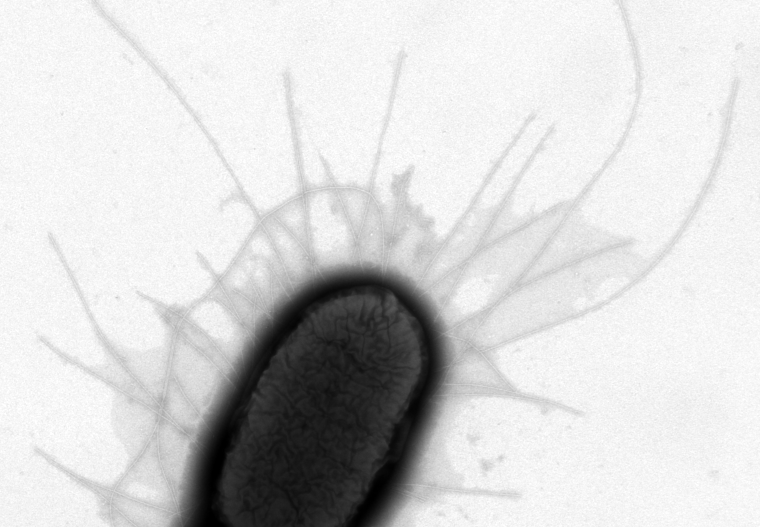


Figure S5. TEM micrograph of a HB101/pHMG93 bacterial cell expressing P-fimbriae. As expected, our data shows that the bacteria express fimbriae at assorted lengths and that they are not overwhelmingly tangled.

# Force spectroscopy measurements

## P-fimbriae in the absence of anti-PapA antibodies


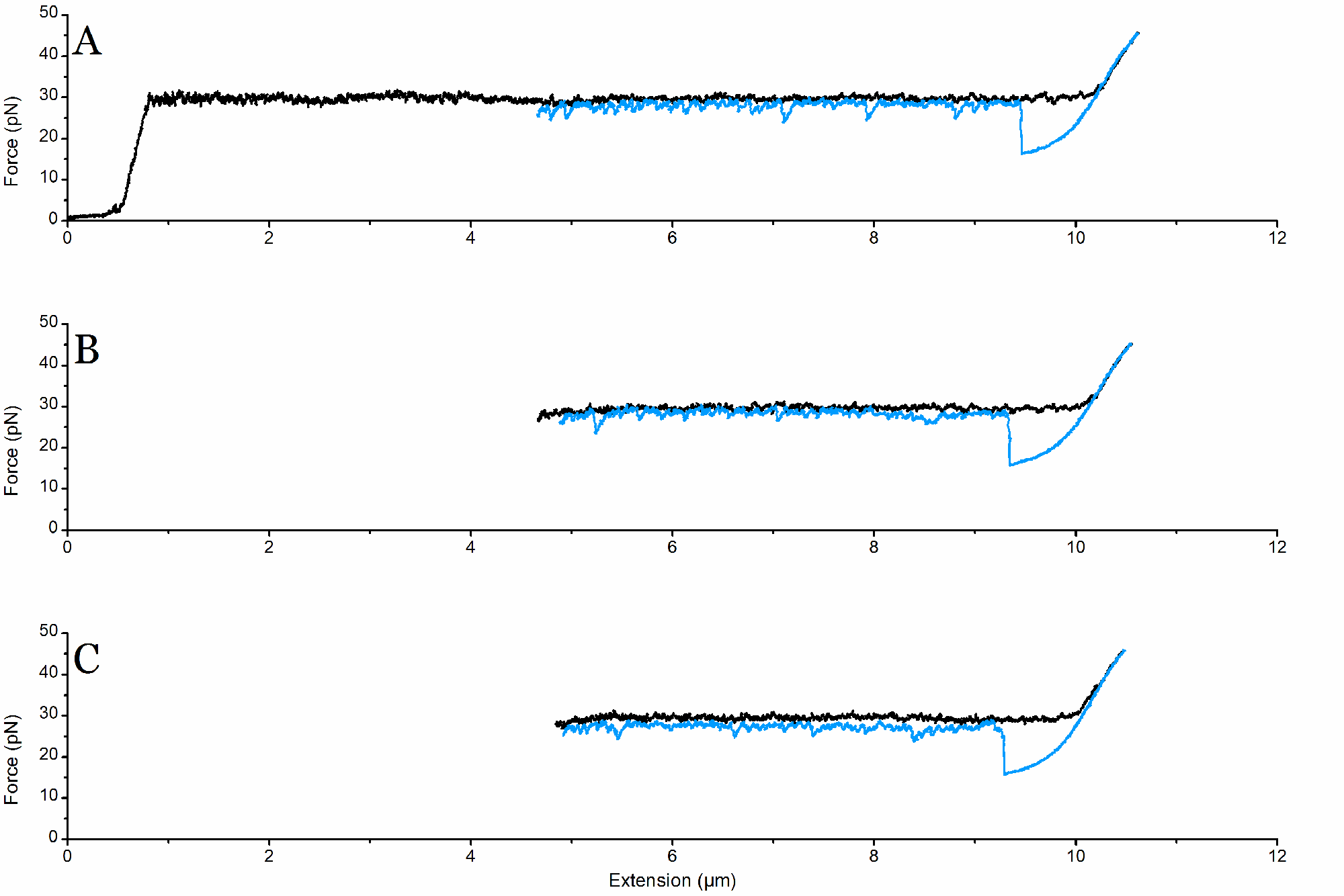


Figure S6. Consecutive force spectroscopy measurement of the same fimbria in the absence of antibodies. Panels A, B and C represent consecutive unwinding (black) and rewinding (blue) cycle for a single P-fimbria in the absence of antibodies.

## P-fimbriae and anti-PapA antibodies at 0.2 µg/ml


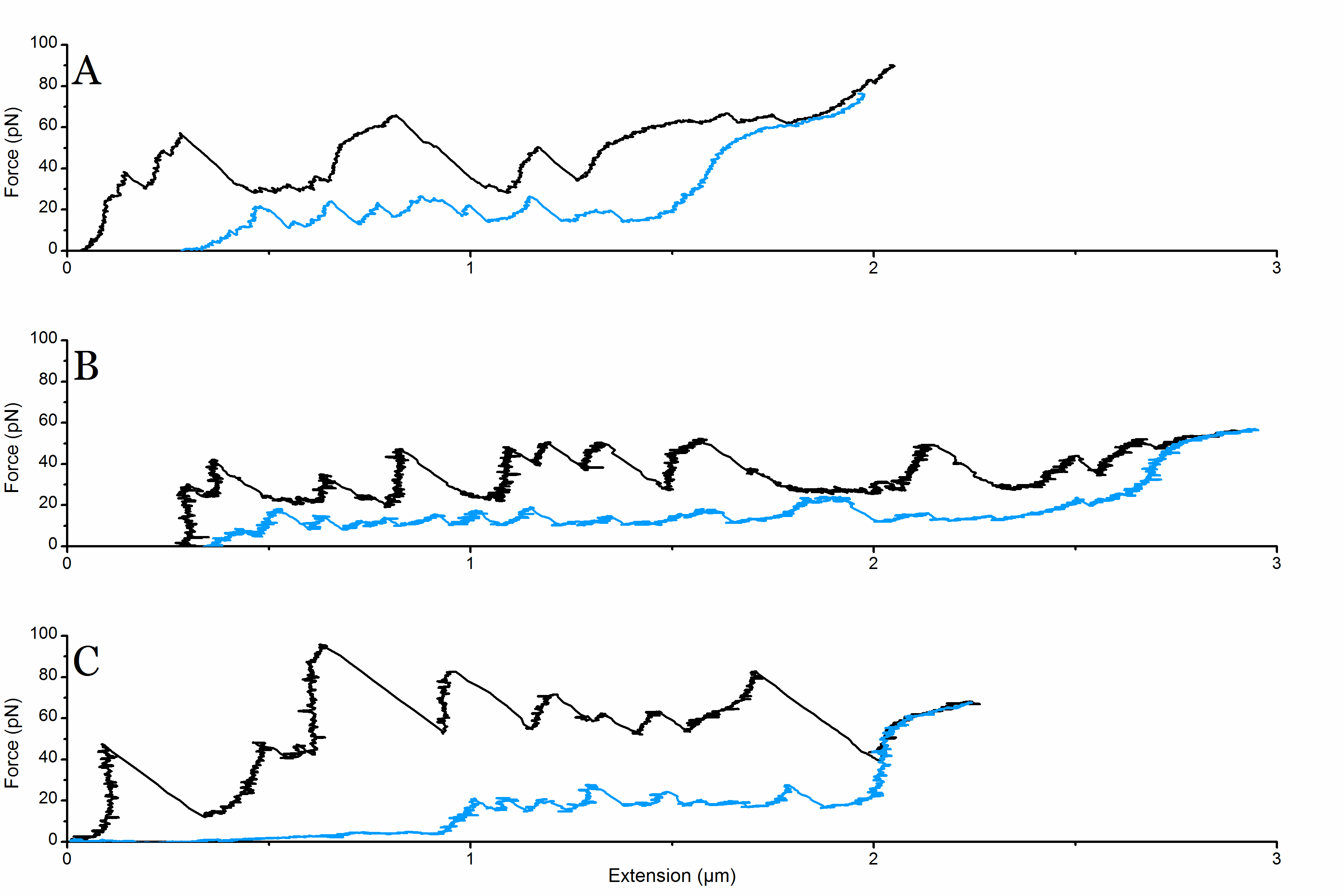


Figure S7. Force spectroscopy measurements in the presence of 0.2 µg/ml anti-PapA antibodies. The panels A, B, and C represent unwinding (black) and rewinding (blue) cycle for three different P-fimbriae in the presence of anti-PapA antibodies (0.2 µg/ml).

## P-fimbriae and anti-PapA antibodies at 2.2 ng/ml


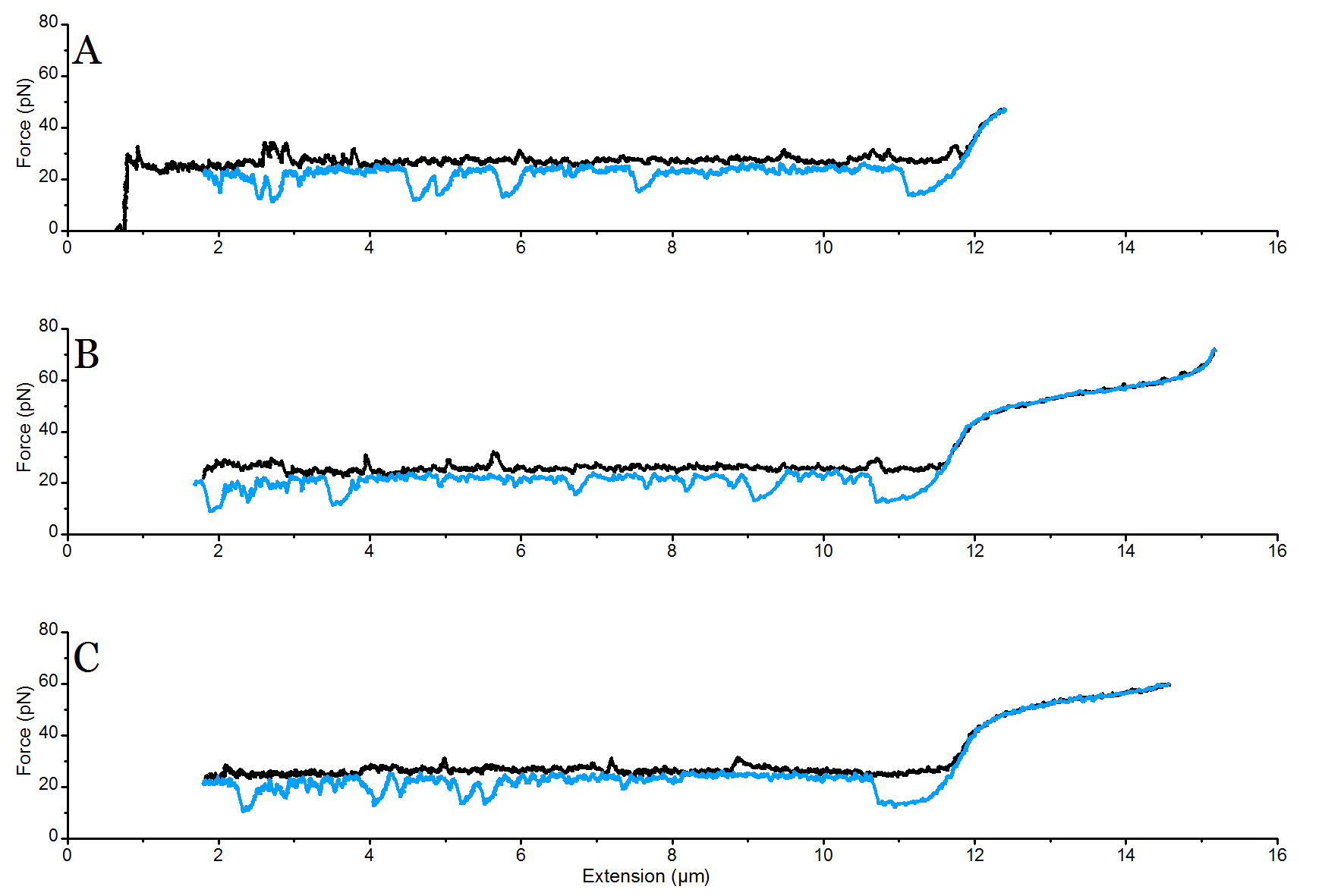


Figure S8. Consecutive force spectroscopy measurements of the same fimbria in the presence of 2.2 ng/ml anti-PapA antibodies. Panels A, B, and C represent consecutive unwinding (black) and rewinding (blue) cycles for a single P-fimbria in the presence of anti-PapA antibodies (2.2 ng/ml).

## P-fimbriae and anti-OmpA antibodies at 0.2 µg/ml


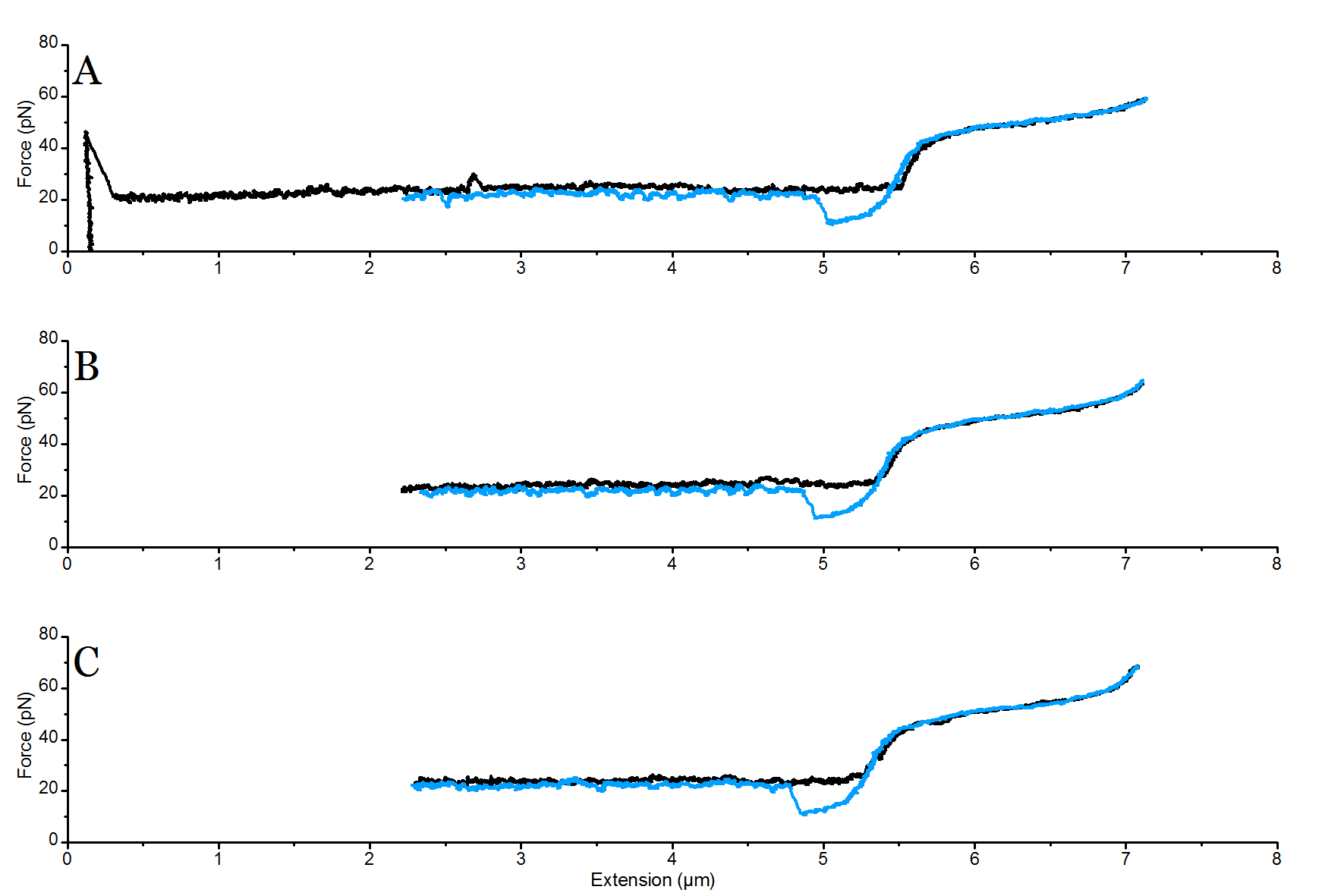


Figure S9. Consecutive force spectroscopy measurements of the same fimbria in the presence of anti-OmpA antibodies. Panels A, B, and C represent consecutive unwinding (black) and rewinding (blue) cycles of P-fimbria in the presence anti-OmpA antibodies.


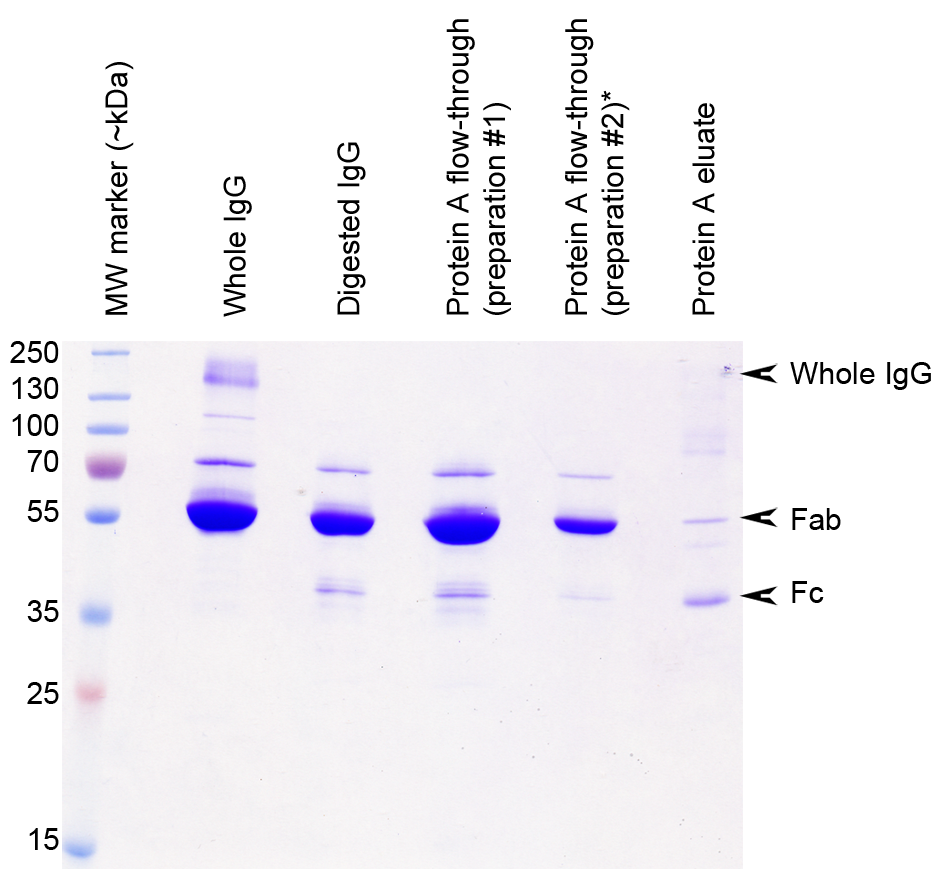


Figure S10. Preparation of Fab fragments. Fab fragments of the rabbit anti-PapA Ab (sniffeV+IV wk, 3.3mg) were prepared using the Fab Preparation Kit from Pierce (44985). In brief, whole IgG was digested with Papain and Fab fragments were purified using Protein A (<http://www.piercenet.com/instructions/2162088.pdf> for detailed procedure). Whole IgG, Fab and Fc fragments were analyzed by non-reducing and non-boiled SDS-PAGE (12%), and stained with coomassie blue. The whole IgG band was absent in Papain digested and Protein A flow-through (purified Fab) fractions, Protein A eluate primarily contained Fc fragments and small amounts of undigested IgGs. Fab fragments from preparation #2 were then used to study the biomechanical responses of P-fimbriae in the presence of Fab fragments.

## P-fimbriae and Fab fragments at 0.2 µg/ml


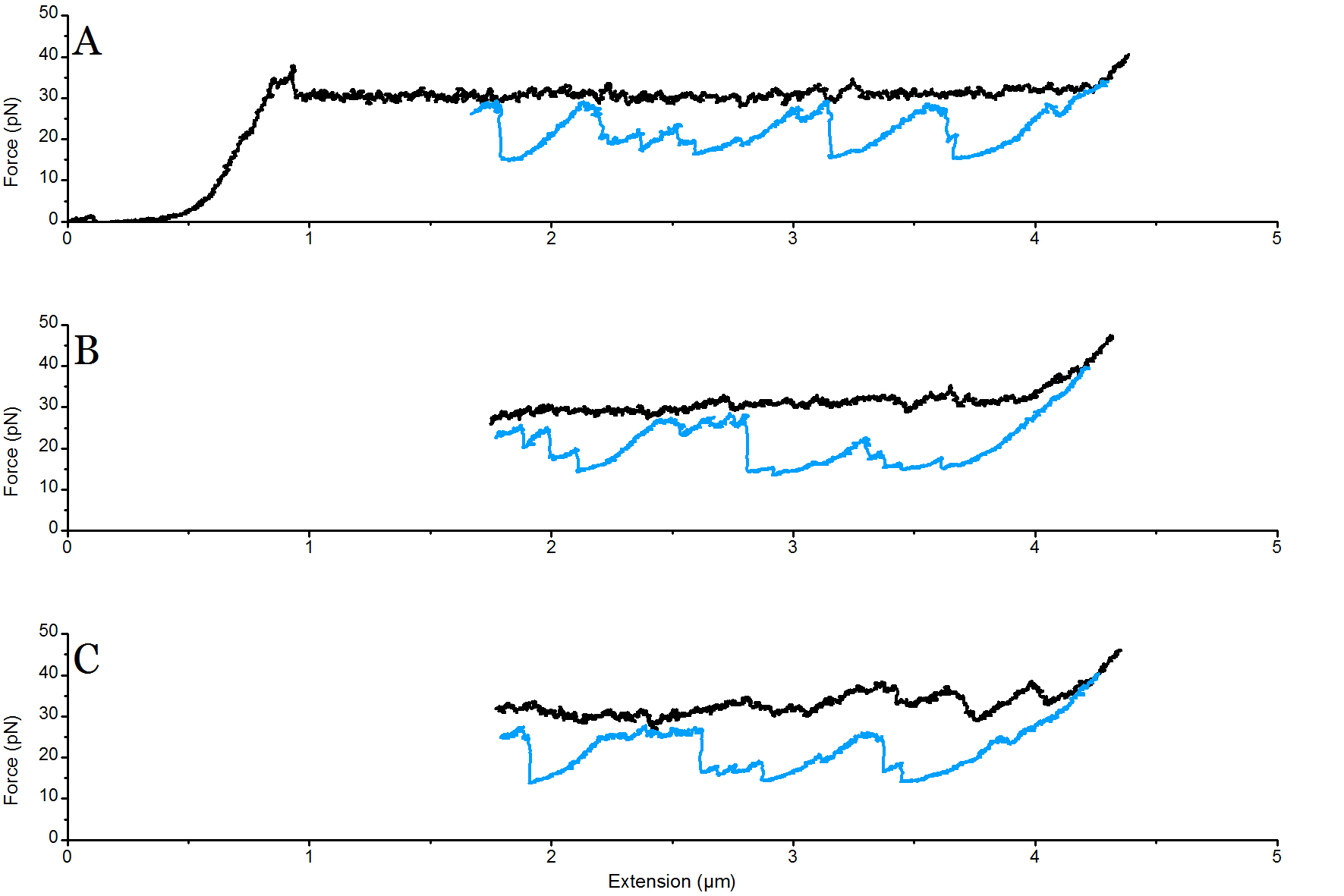


Figure S11. Consecutive force spectroscopy measurements of the same fimbria in the presence of Fab fragments. Panels A, B and C represent consecutive unwinding (black) and rewinding (blue) cycles of a P-fimbria in the presence 0.2 µg/ml Fab fragments from anti-PapA antibodies. The Fab concentration was determined using a Nanodrop.

## Multiple P-fimbriae force-extension


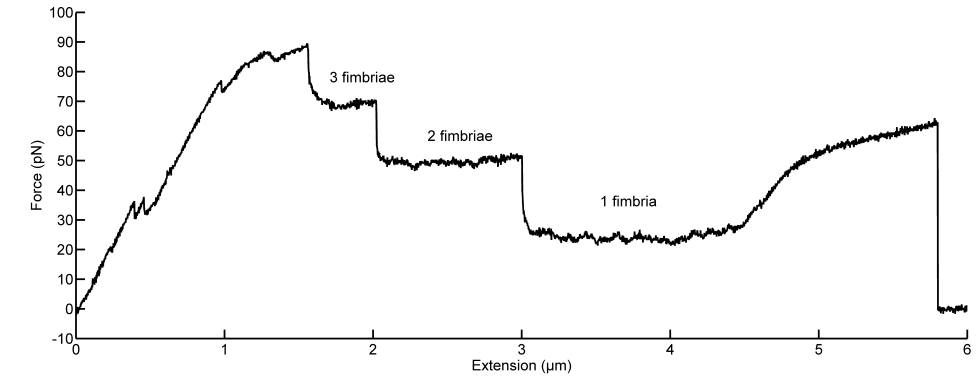


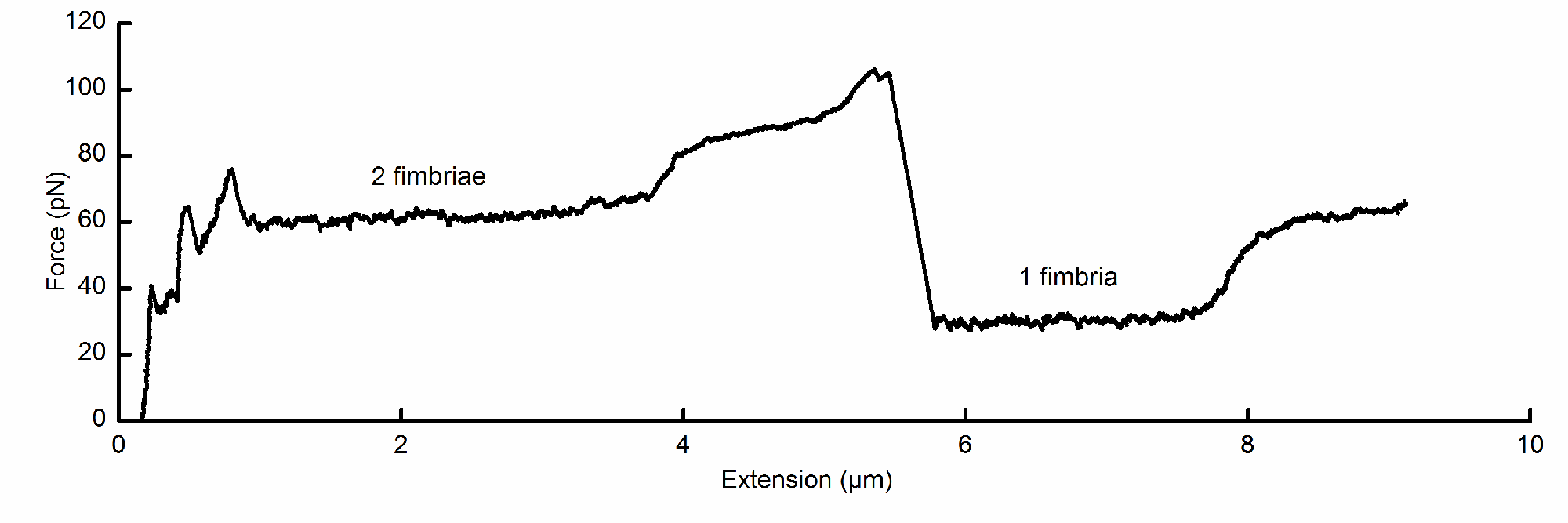


Figure S12. Two force spectroscopy data curves from different measurements showing multi-fimbriae attachment. The data shows the force response when several fimbriae, given by *n*, are attached to a probe bead. Discrete unwinding levels are then given by *n* x 30 pN. For example, the bottom curve has two levels representing two and one fimbriae attached, one at ~60 pN and the other at ~30 pN.

# Model parameters used in the individual sticky-chain model

The following model parameter values were used in the fit presented in figure 3D; energy difference between the two states, $V^{0}=5.9\text{ }kT$, the elastic constants $k_{B}=150\text{ pN/nm},\text{ }k_{C}250\text{ pN/nm}$, the distance to the transition barrier, $x^{t}=0.7\text{ }nm$, and between the states, $\Delta x=0.86\text{ }nm$. A detailed description of the model is given in refs ^1,2^.

# Calculation of the average number of layers clamped using force peak data

The force *F* measured by optical tweezers is given by,

$F=k\Delta x$ (1)

where *k* is the trapping constant (~140 pN/µm) and $\Delta x$is the displacement of the bead in the trap. Thus, the force drop seen in the force-extension data, see Fig. 5A for an example can directly be related to the instantaneous length increase of a fimbriae$\Delta L$, i.e., $\Delta L=\Delta x$. When an antibody binds the *n^th^* and *n+*7*^th^* subunits (8 subunits) and one arm suddenly detaches in an experiment, the fimbria is instantaneously elongated. The elongation length is given by the number of subunits that open, times the bond opening length $\Delta x_{AB}$ of a subunit, which is ~3.5 nm for PapA ^1^. The corresponding force peak that we would expect in an experiment would then be,

$$F_{\text{force peak}}=k\Delta x=140\text{pN/µm}\cdot8\text{subunits}\cdot0.0035\text{pN/subunit}\approx4\text{pN}$$

The bond opening length $\Delta x_{AB}$of PapA subunits was estimated by fitting a theoretical model to experimental data. However, the bond opening length of the FimA subunits in type 1 fimbriae, which is similar to that of P fimbriae, was directly measured by atomic force microscopy by Forero et al ^3^. It was found in these experiments that the bond opening length for these subunits was slightly larger, i.e., 5.0 ± 0.3 nm. By using this result the corresponding force would be slightly higher ~6 pN.

# Movie caption

**Supplementary Movie 01.** 3D rendering of confocal images showing peripheral localization of P-fimbriae (green) and intracellular localization of nucleoid (blue).

**Supplementary Movie 02.** Modeling conditions for finding the possible attachment positions of an antibody on the shaft of a P-fimbriae. The blue cylinder represents one of the antibody arms attached to the *n*^th^ subunit. The second arm is restricted to attach to other subunits within the volume of the sphere.

# References

1. Andersson, M., Fällman, E., Uhlin, B. E. & Axner, O. A sticky chain model of the elongation and unfolding of Escherichia coli P pili under stress. *Biophys. J.* **90,** 1521–34 (2006).

2. Andersson, M., Fällman, E., Uhlin, B. E. & Axner, O. Dynamic force spectroscopy of E. coli P pili. *Biophys. J.* **91,** 2717–25 (2006).

3. Forero, M., Yakovenko, O., Sokurenko, E. V, Thomas, W. E. & Vogel, V. Uncoiling mechanics of Escherichia coli type I fimbriae are optimized for catch bonds. *PLoS Biol.* **4,** 1509–1516 (2006).
